# Mechanisms of Creativity Differences Between Art and Non-art Majors: A Voxel-Based Morphometry Study

**DOI:** 10.3389/fpsyg.2018.02319

**Published:** 2018-12-11

**Authors:** Tan Xurui, Yu Yaxu, Li Qiangqiang, Mao Yu, Zhou Bin, Bao Xueming

**Affiliations:** ^1^School of Communication of East China Normal University, Shanghai, China; ^2^Department of Psychology, Southwest University, Chongqing, China; ^3^Key Laboratory of Cognition and Personality, Ministry of Education, Chongqing, China; ^4^College Students Psychological Counseling and Health Center, Party Committee Student Work Department, East China University of Technology, Nanchang, China; ^5^Institute of Cultural and Creative Industry of Shanghai Jiao Tong University, Shanghai, China; ^6^School of Sports and Health of East China Normal University, Shanghai, China

**Keywords:** creativity, art major students, non-art major students, GMV, MFG, ACC

## Abstract

Creativity is considered the ability to generate new ideas or behaviors, an ability that have diverse expressions in different human groups, such as painters and non-painters. Art major students require more creative activities than non-art students do. In this study, we plan to explore the figural creativity abilities of art major students and whether these students exhibited higher figural creativity scores and why their brain structure of gray matter are lower which may benefit from their professional training relative to non-art majors. Therefore, in this study, we use voxel-based morphometry (VBM) to identify different behavioral and brain mechanisms between art major students and non-art major students by using the figural Torrance Test of Creative Thinking. Our results showed that the TTCT-figural (TTCT-F) scores of art majors were higher than those of non-art majors. The TTCT-F score of art major students and practicing and study time have positive correlations which means art major’s figural creativity score benefit from there art professional training in some degree. Subsequently, the interaction analysis revealed that the TTCT-figural scores of art majors and non-majors exhibited significant correlations with the gray matter volumes (GMV) of the left anterior cingulate cortex (ACC) and the left medial frontal gyrus (MFG). While the simple slope analysis showed that art majors, compared with non-art majors, exhibited a marginal significantly positive association with the left ACC and MFG, non-art majors exhibited a significantly negative association with the left ACC and MFG. Overall, our study revealed that people who major in artistic work are more likely to possess enhanced figural creative skills relative to non-artistic people. These results indicated that professional artistic programs or training may increase creativity skills via reorganized intercortical connections.

## Introduction

There have been many different theories of creativity until recently, and the general idea regarding creativity is that it refers to the generation of original, novel ideas through mental habits of thinking (Torrance, 1966, 1988; Guilford, 1967; Guilford et al., 1978; Ruscio et al., 1998; Howard-Jones et al., 2005; Chavez-Eakle et al., 2007; Gibson et al., 2009; Storm et al., 2011). Simonton argued that creativity is certainly the most important and common of all human activities, being seen as an attribute for people to possess (Simonton, 2000). Torrance also said that creativity is seen as contributing original ideas, distinct points of view and new angles to looking at problems (Torrance, 1988). In recent decades, creativity research mostly began with Guilford who claimed creativity is consisted of convergent thinking and divergent thinking (Guilford, 1967). While Divergent thinking includes thinking out solutions to a problem, which means multiple solutions. An example like Alternate Uses Task where individuals aer asked to think of as many possible ways to use an object, such as brick (e.g., “House build”). Solutions should be novel and appropriately useful (Guilford et al., 1978). Several studies have probed the neural mechanisms of creative-thinking abilities, including those utilizing functional magnetic resonance imaging (fMRI), positron emission tomography (PET), measurements of regional cerebral blood flow (rCBF), and electroencephalograms (Heilman et al., 2003). Many psychometric instruments have been used to develop tests of people’s creative abilities, among which, one of the most common ones is Torrance Test of Creative Thinking (Torrance, 1966).

Individual creative abilities differ, dividing people into diverse groups according to the work they do. In previous studies, researchers usually divided people into two groups according to the TTCT sub or total scores and then analyzed this phenomenon. An fMRI study revealed that TTCT-figural (TTCT-F) scores were prominently higher in high group than those in low group (Jiao et al., 2017). A creativity study revealed that creative scores were higher in high group than those in the low creativity group (Li et al., 2017). An innovative study of improvisation intervention showed better abilities of divergent thinking and become more creativity after intervention, suggesting improvisation as one of the simple and art-based interventions would have formal field of common benefits for creative cognitive processes. In addition, these findings indicated primary school children could make better use of subsistent art education provision that could offer an efficient way to obtain creativity abilities (Sowden et al., 2015). Participants who accept creative training programs’ content with exercises or activities relative to control group shown significantly increase in their creative thinking abilities (Ulger, 2016). In addition, a study of neural networks about expert and novice group that higher creativity scores showed by the expert subject were compared the novice groups (Kowatari et al., 2009).

As mentioned, studies have also divided people according to their skills that have been mastered such as professional dancers and novice dancers or musicians and novices, and creativity (Byron and Khazanchi, 2011). An EEG study about generating of alternative uses between professional and novice dancers found that in posterior parietal brain regions, professional dancers show stronger alpha synchronization than novice dancers did. During improved dance, greater right-hemispheric alpha synchronization showed in professional dancers than novice did (Fink et al., 2009). In a study of musicians and non-musicians, participants used a creativity task named novel divergent thinking task to explore and generate uses for them alone and in combination with one another. The results indicates that musicians generate a greater number of “uses” than the non-musicians in both single objects and combinatory uses (Gibson et al., 2009). Another study about musicians conclude that the brain regions of prefrontal and paralimbic areas, including insula are related to network integration, these areas mostly related to cognitive, motivational, and emotional processes. Specifically highly creativity ability’s individual completed their creations based on the original rhythm, the activation of brain regions include bilateral prefrontal regions and right insula. While low creativity ability’s individual completed their creations, the changes only express in original musical patterns (Villarreal et al., 2013). In an EEG study of gifted, intelligent, creative, and average individuals, in solving creative problems, highly creative individuals were revealed less mental activity than average individuals were. In brain areas interaction, creative individuals also showed better than gifted ones, who exhibited substantial decoupling of brain areas when solving ambiguous problems (Jaušovec, 2000). The prefrontal cortex (PFC) is also a pivotal structure that is involved in divergent thinking, which is a crucial factor of creative innovation (Heilman et al., 2003; Ghacibeh and Heilman, 2013).

Recently, several studies have proven differences in figural creative processes between painters and non-painters (Eindhoven and Vinacke, 1952; Wallach and Kogan, 1964; Berlin and Kay, 1991; Wolff and Lundberg, 2002; Bhattacharya and Petsche, 2005; Burch et al., 2006; Bos et al., 2006). Eindhoven used naturalistic methodology to observe the behavior of painters and non-painters working on sketching paper. The researchers observed painters completing their works using four stages, namely, the gradual, experimental, concentration, and reorganization stages. In contrast, non-painters displayed no staging strategies (Eindhoven and Vinacke, 1952). Moreover, reports by Kay indicated that the reaction times of painters during problem-solving tests are longer than those of non-painters. Wolff conducted an objective test wherein they found that art students had significantly worse phonological skills and less reported risks associated with dyslexia than non-art students. Burch used the instances task to test creative ability and found that visual painters scored higher than non-painters on uniqueness. Likewise, Bhattacharya and Petsche demonstrated differences in the patterns of functional integration between cortical regions during the mental creation of drawings created by painters and non-painters. An EEG study also showed that creative ability in a figural creativity task was associated with significantly stronger desynchronization of upper alpha power, indicating high figural processing demands (Rominger et al., 2018). An fMRI study showed that TTCT-F scores in the high group were associated with several brain regions, including the left temporal cortex, left precuneus, left thalamus, and right fusiform gyrus, right posterior occipital cortex. In contrast, TTCT-F scores in low group were associated with the right posterior cingulate cortex, ventral medial prefrontal cortex, right dorsal frontal cortex, and right inferior parietal lobule (Jiao et al., 2017). Another fMRI study of artists and creative individuals also demonstrated significantly strong functional connectivity in the right angular gyrus, bilateral inferior frontal gyrus, and bilateral superior frontal gyrus (de Souza et al., 2010).

As mentioned above, the creativity skills related to special and normal domains are still controversial, particularly the basic differences among brain regions. Therefore, in this study, art and non-art majors underwent structural MRI scans after they performed a figural creative thinking task. Creativity scores were then assessed using the TTCT-F and related gray matter volume changes were observed during brain scanning. We expected that the figural scores of the art major students would be higher than those of the non-art major students due to the long professional training regarding art. Moreover, interaction effects of the GMV in the brain and TTCT-F scores were expected: the GMVs of specific cerebral areas were expected to have significant effects [e.g., the medial frontal gyrus (MFG), anterior cingulate cortex (ACC), and superior temporal gyrus (STG)] on figural creativity within the brain activation of art majors. The results of this study may provide insight into the cultivation of creative education among art major students in the aspect of figural creativity brain activation of art majors.

## Materials and Methods

### Participants

Eighty painting subjects (40 art majors and 40 non-art majors; (33 men, aged 18–22 years, mean = 20.24 years; 47 women, aged 18–24 years, mean = 20.23 years) participated in this research as part of our project investigating associations among brain imaging, intelligence, and the TTCT. Inclusion criteria for the 40 art major group were as follows: current studying and professional paint training time for more than 1 years and practiced for more than 1 h each day. Majors included sculpture, traditional Chinese painting, watercolor, sketch and oil painting. 40 non-art major students had no painting training beyond the regular curricular exposure to painting during the kindergarten to university years. All participants were right-handed, had normal vision, had no history of psychiatric or neurological illness and were undergraduates at Southwest University. After providing written informed consent, participants received payment for their time.

### Assessment of Creativity

The TTCT (Torrance, 1966), which was used to assess creativity (i.e., divergent thinking ability), consists of verbal, figural, and auditory tests (Huang et al., 2013). In our study, the TTCT-figural (TTCT-F) test was used to measure individual divergent thinking ability (Torrance, 1988; Carson et al., 1994; Kim, 2006). We employed the TTCT-F test to evaluate both art and non-art students. Three factors were concluded in TTCT-F total score: flexibility (the number of different types of answers, demonstrating the ability to switch conceptual fields); fluency (the number of relevant and meaningful answers, which are relative to the ability to generate a number of pictures or objects); and originality (the number of unique ideas, which reflects the ability to produce unique or uncommon answers) (Kim, 2006). Heausler and Thompson (1988) declared that the TTCT-F total score is highly associated with scores of the three parts (fluency, flexibility, and originality) and that the scores of the three parts are highly associated with each other (The correlation coefficient between these three parts > 0.81). Further, strong correlations among the three components of the TTCT do not provide meaningfully different data; thus, the total TTCT score used as an accurate index of creativity (Heausler and Thompson, 1988).

### Assessment of General Intelligence

To assess mental capacity, all participants finished Chinese-revised edition of the Combined Raven’s Test (Li & Chen), which has a reliability coefficient of 0.92 (Li and Chen, 1989; Ming, 1989). The CRT contains Raven’s standard progressive matrix and Raven’s colored progressive matrix, which includes 72 items that were revised by the Psychology Department of East China Normal University in 1989. The CRT test score (number of correct answers given in 40 min) was used as a psychometric index of personal intelligence. In line with standard practice, this study focused on the total score of the test (Takeuchi et al., 2011).

### Image Acquisition

All images were gathered using a 3-T Siemens Trio MRI scanner (Siemens Medical, Erlangen, Germany). High-resolution T1-weighted structural images were collected using a magnetization-prepared rapid gradient echo (MPRAGE) sequence. The parameters were as follows: repetition time = 1900 ms, inversion time = 900 ms, flip angle = 9 degrees, echo time = 2.52 ms, 256 × 256 matrix, 176 slices, 1.0 mm slice thickness, and voxel size = 1 mm^3^ × 1 mm^3^ × 1 mm^3^.

### MRI Preprocessing

All images were processed using SPM8 ^1^ implemented in MATLAB R2014a (MathWorks Inc., Natick, MA, United States). First, every magnetic resonance (Semrud-Clikeman et al., 2016) image was displayed in SPM8 to monitor artifacts and obvious anatomical abnormalities. In addition, VBM was performed with diffeomorphic anatomical registration using exponentiated lie algebra (DARTEL) (Ashburner, 2007). The New Segment Toolbox from SPM8 was applied to every T1-weighted MR image to extract tissue maps corresponding to gray matter, white matter and cerebrospinal fluid in the native space. Using the DARTEL template-creation toolbox, the resliced images of the gray and white matter were then registered to a subject-specific template to improve intersubject alignment. Subsequently, the normalization function in the DARTEL toolbox was used to normalize the individual images of gray and white matter to the MNI space (1.5-mm isotropic voxels). Finally, the gray and white matter maps of each subject were warped using their corresponding smoothed (8-mm full-width at half-maximum (FWHM) Gaussian kernel), reversible deformation parameters to the custom template space and then to the MNI standard space. Gray matter volume (GMV) images were modulated by calculating the Jacobian determinants derived from the special normalization step and by multiplying each voxel by the relative change in volume.

### Statistical Analysis

In order to test our hypothesis of art major students’ TTCT-F scores are higher than non-art major students may result in professional training mostly than other reasons, we collect art major student’s professional paint training duration and practicing time each day last month. Then correlated to the TTCT-F score of art major students. It is shown that these two variances are significant positive with art major student’s TTCT-F score. This shows a certain degree of professional training of art may enhance their creativity skill of figural.

We investigated whether there was an interaction effect between the majors and figural creativity on brain structure. At the whole-brain level, a voxelwise analysis of covariance (ANCOVA) was performed using the full factorial option in SPM8, in which major was defined as a group factor. Age, sex, general intelligence, total score on the TTCT-F, and total GMV were entered as covariates; the total score on the TTCT-F underwent interaction analysis with majors using the interactions option in SPM8, which facilitated investigation of the interaction effect between majors and the covariates of brain structure. These interaction effects were assessed using t-contrasts, and the t-contrasts were performed by setting the voxelwise intensity threshold at *p* < 0.005, as determined by the non-stationary cluster extent correction (NS) in SPM.

## Results

### Descriptive Statistics

The demographic data and behavioral results are shown in Table 1. Art majors showed significantly higher total TTCT-figural scores than the non-art majors (*p < 0.01, t = 3.87,* two-tailed *t*-test). Intelligence, as measured using the CRT, was not significantly different between majors (*p* > 0.05, *t* = -1.052, two-tailed *t*-test).

**Table 1:** Demographic data and behavioral results.

Measures	Art majors (*n* = 40) Means	SD	Non-art majors (*n* = 40)Means	SD
TTCT (total)^∗∗∗^	71.750	20.208	55.633	16.838
CRT	64.725	4.867	65.775	4.022
Age	20.375	1.030	20.100	1.033


*TTCT, figural torrance tests of creative thinking; CRT, the combined Raven’s test. ^∗∗∗^*p* < 0.01.*

### Correlation Analysis Between Behavioral Measures

The correlation between behavioral measures of TTCT-F score and practicing time are significantly positive, which means the higher TTCT-score participants get, the more practicing time they need. On the other hand, the TTCT-F score of art major students and professional paint training duration had marginal, significant positive correlations, which also certified TTCT-F score of art major students are owing to professional art practice mostly, see Table 2.

**Table 2:** Correlation analysis between behavioral measures.

	TTCT-F (total)	Study time	Practicing time
TTCT-F (total)	–	0.076^∗^	0.003^∗∗∗^
Training duration		–	0.609
Practicing time			–


*^∗∗∗^p < 0.001; ^∗^p < 0.01.*

### Interaction Effect of Major and Creativity on Regional GMVs

**FIGURE 1:**
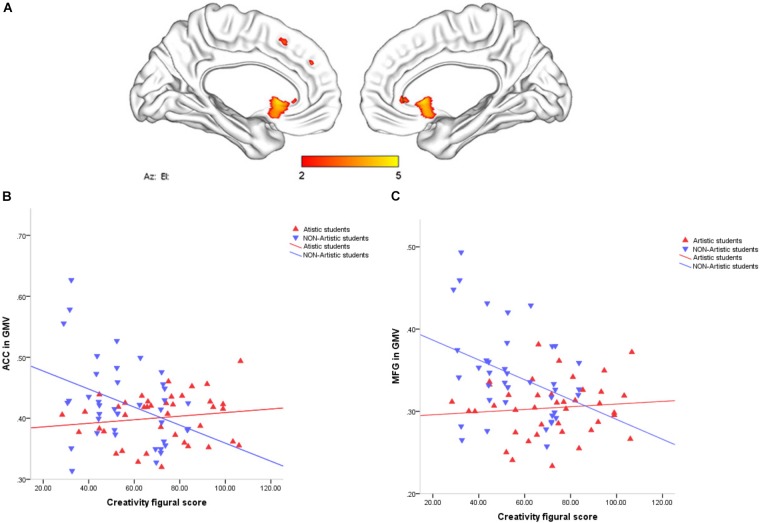
**(A)** The interaction effect of TTCT-F scores in art majors and non-art majors and the GMVs of the left anterior cingulate cortex (ACC) and left medial frontal gyrus (MFG) (peak voxel MNI coordinate: -2, 18, -2, *T* = 4.5; -11, 50, 19, *T* = 4.2), corrected with the non-stationary cluster extent correction (NS) at the whole-brain level. **(B**,**C)** Figural creativity scores of the art majors demonstrated a marginal significant positive correlation with the GMVs of the left ACC and MFG (*p* > 0.05), whereas a significant negative correlation was observed in the non-art majors (*p* < 0.05).

An interaction effect between figural creativity scores among art majors and non-art majors was shown on GMV in the left anterior cingulate cortex (ACC) and left medial frontal gyrus (MFG) (peak voxel MNI coordinates: -2, 18, -2, *T* = 4.5; -11, 50, 19, *T* = 4.2), see Figure 1A and Table 3, corrected with the non-stationary cluster extent correction (NS) at the whole-brain level. In particular, the figural creativity scores of the art majors had marginal, significant positive correlations with the GMVs of the left ACC and MFG (*p* > 0.05), while those of the in non-art majors ha significant negative correlations (*p* < 0.05), see Figures 1B,C. To further validate our findings, we conducted independent samples *t*-tests with active regions as seeds (see Table 4). The results supported our hypothesis that the voxel sizes of art majors are smaller than those of non-art majors.

**Table 3:** The interaction effect of brain regions significantly correlated with creativity.

Item	Clustersize	H	Peak Tvalue	Peak MNI coordinate	Brain regions
TTCT-F creativity (NS Corrected *p* < 0.005)	45	L	4.2	-11 50 19	Medial frontal gyrus
	614	L	4.5	-2 18 -2	Left cingulate cortex


*MNI, Montreal Neurological Institute; H, hemisphere; L, left; NS, non-stationary cluster extent correction for SPM.*

**Table 4:** Independent sample *t*-test analysis with active regions as seeds.

Seed ROI	Art majors (*n* = 40) Means	SD	Non-art majors (*n* = 40) Means	SD
Left MFG^∗∗∗^	0.425	0.0682	0.344	0.055
left ACC^∗^	0.401	0.0401	0.304	0.034


*MFG, medial frontal gyrus; ACC, anterior cingulate cortex. ^∗∗∗^*p* < 0.01; ^∗^*p* > 0.05 (marginal significance).*

## Discussion

In this study, we investigated associations between brain structures and individual figural creativity. Our behavioral results showed that art majors had significantly higher total TTCT-figural scores than non-art majors. Further, the VBM analysis results showed that figural creativity scores were related to GMVs of the left ACC and MFG. Specifically, in a simple slope analysis, a marginal, significant positive effect occurred in art majors and figural creativity in the GMV regions, while in non-art major, a significantly negative relationship was shown between figural creativity and the GMVs of specific brain regions. These differences indicated that art major students likely spend more time engaging in professional courses and have more chances to engage in creative activities, such as novel painting or original works. We figured that the impact of this factor on the results of the test would be significantly different between major and non-major students.

In the present study, the TTCT-figural scores of art majors were higher than those of non-art majors. Some researchers have postulated that individual differences in creativity skills are modulated by certain cognitive skills (Fischer and Rose, 1998; Gibson et al., 2009). Brain plasticity is a theme that has been verified in many studies (Chugani, 1994; Kolb and Whishaw, 1998; Johansson, 2000; Cotman and Berchtold, 2002; Ungerleider et al., 2002; Mahncke et al., 2006; Smith et al., 2009; Chan et al., 2016). Cotman clarified that brain-derived neurotrophic factor and other growth factors can increase through conscious exercise, it also can increase the level of stimulate neurogenesis, increase resistance when encounter brain insult, and improve learning ability and mental performance. While solving divergent thinking tasks, subjects with high scores had lower EEG dimensions than the subjects with low scores, particularly in frontal cortical areas (Mölle et al., 1996). Prior research indicates that the adolescent brain is indeed sensitive to the effect of training of different cognitive functions including working memory and mathematical skills (Qin et al., 2004; Jolles et al., 2010). This means that people with different knowledge backgrounds solve problems in different ways (Schoenfeld, 2014). A creativity study of imagery involving high- and low-imagery subjects showed some differences in performance on cognitive tasks obtained in the studies (Campos and Pérez, 1989). In a training study, in the improvisation group subjects express higher creativity score, which means better divergent thinking ability in some degree (Sowden et al., 2015). Another study of topological organization and creativity showed that TTCT-F scores were significantly higher in the high group relative to the low group. A spontaneous improvisation and figural creativity which pictionary-based fMRI study showed that drawings is a good form with a given word were also creative (Saggar et al., 2015). Regarding the neurobiology of creativity, the creative strength of high groups mostly scored high (Carlsson et al., 2000). Urban stated that the training and learning with an open structure are highly important for creative education, which encourages non-conformist behavior, including problem solving and divergent thinking (Urban, 1995). Another study of creativity showed that lecturers could choose learning outcomes as open-ended serial constructions to encourage creativity in students (Giloi and du Toit, 2013). An EEG study showed that figural field especially creative ideation is associated with many specific task and sensory-based visual mental operations (Marks and Isaac, 1995; Petsche et al., 1997). From the above studies, we may conclude that subjects with high figural creativity ability are mainly associated with executive control, attention, and memory retrieval networks in functional connectivity.

In the analysis of the brain, our results showed that the TTCT-figural scores of art majors have a marginal significant positive effect on the GMVs of the left ACC and MFG. In terms of the marginal significant positive effect of figural creativity on the GMV in the left ACC, as mentioned before, divergent thinking has consistently been shown to be related to widespread brain regions, such as the PFC and anterior cingulate cortex (Wu et al., 2015; Sun et al., 2016; Shi et al., 2018). A meta-analysis which activation likelihood estimation was used to detect divergent thinking of neuroimaging found that lateral prefrontal cortex, posterior parietal cortex, preceneus, anterior cingulate cortex, and temporal gyrus were the key regions (Wu et al., 2015). Additionally, our team’s work regarding creativity training found that the dACC changes between pre- and posttests, both in terms of functional activity and gray matter (Sun et al., 2016). Furthermore, a combined VBM and resting-state functional connectivity study of creative achievement indicated that the ACC and bilateral frontal-insular cortex are negatively correlated (Sridharan et al., 2008; Chen et al., 2014). An fMRI study showed that between creative and uncreative in participants the activation located in bilateral medial frontal gyri and the left anterior cingulate cortex which belong to prefrontal areas (Howard-Jones et al., 2005).

As for the interaction results of the left MFG between majors and VBM, the fMRI study also showed that there were increases in the medial frontal gyri (MFG). The left ACC and MFG in the left hemisphere of the brain may have a facilitatory effect on art major students. The MFG, known as the “inhibitory area,” has been verified in tasks that require associations of stimulus-response (Kreitzer and Malenka, 2008; Brass et al., 2009; Dwyer et al., 2009; Hill and Miller, 2010; Westerhausen et al., 2010; Gautam et al., 2011; Kozasa et al., 2012). Westerhausen also clarified that the MFG might be associated with top-down control of attentional processes. The MFG may also play a part crucial role in working memory during the manipulation of actively maintained information (Bunge and Zelazo, 2006; Woodward et al., 2006; DeYoung et al., 2010). These findings suggest that the reduced GMV in the MFG revealed in the present study might be associated with reduced inhibitory control, which may be associated with particular characteristics of higher trait creativity, such as challenge and risk taking.

We concluded that the educational environment of art majors is largely different from that of non-art majors. This view has also been confirmed by previous researchers (Golomb and Galasso, 1995). For example, art majors are typically tested on visual information and on the long-term practice of art skills that are required to generate creative outputs, which can better exercise your brain (Cotman and Berchtold, 2002). These behaviors, such as professional art training for art major students, may enhance divergent thinking. To some degree, a reduced left hemisphere itself inhibits. Art majors engage more in professional training skills than non-art majors, skills that are linked to improving visual orientation abilities to determine the correct direction to be taken and the ability to make complicated hand movements, which can enhance their cranial nerve more effectively (Griffin, 2017). Moreover, some functional studies revealed cingulate involved in internal selection and frontal region engagement relevant to task complexity (Starchenko et al., 2003). These differences in the interaction between the creativity scores of the two types of college majors could be explained on the one hand by the professional learning experience of art students. On the other hand, the results suggested that creativity could be promoted in art majors by reducing the level of inhibition in the brain hemisphere and task execution. A further analysis in our study regarding ROI value *t*-tests revealed that the values of art majors were smaller than those of non-art majors. However, in general, non-art major students rarely engage in designing art or in creative activities relative to art majors. In particular, the left ACC and MFG of art majors are smaller than those of non-art majors. As one of the forms of information representation, visual mental images involve top-down information processing based on experience. The idea is that the material of the thinking process, of which a person has different expression forms of information in the brain, is more suitable for generating creative thinking if understanding of composition occurs.

## Conclusion

In the present study, the behavioral results indicated that the TTCT-figural score of art majors are higher than non-art majors. In addition, this fMRI study revealed that the TTCT-figural scores of art majors exhibited a marginal significant positive correlation with left ACC and MFG GMVs. These results show that, in art majors with high figural creativity, long-term exercise of artistic training may broaden their creativity skills and enhance their brain plasticity more than in non-art majors. Prolonged art training broadens the ability of the brain to think openly and may, in some ways, reduce the inhibitory effect on the right hemisphere.

## Ethics Statement

The protocol was approved by Southwest University Brain Imaging Center Institutional Review Board and written informed consent.

## Author Contributions

All authors listed have made a substantial, direct and intellectual contribution to the work, and approved it for publication.

## Conflict of Interest Statement

The authors declare that the research was conducted in the absence of any commercial or financial relationships that could be construed as a potential conflict of interest.

## Footnotes

1 https://www.fil.ion.ucl.ac.uk/spm/
